# Peer Review of “Technologies to Support Assessment of Movement During Video Consultations: Exploratory Study”

**DOI:** 10.2196/32265

**Published:** 2021-09-24

**Authors:** Radu Ciorap

**Affiliations:** 1 Department of Biomedical Sciences Faculty of Medical Bioengineering Grigore T. Popa University of Medicine and Pharmacy Iasi Romania

**Keywords:** tele-rehabilitation, video-consultations, assessment of movement, eHealth, technology, desktop robots, wide-angle webcams, physical health, rehabilitation, remote, assessment, assistive technology, evaluation, framework, webcam, telehealth, robots

*This is a peer-review report submitted for the paper “Technologies to Support Assessment of Movement During Video Consultations: Exploratory Study.”*

## Round 1 Review:

The paper [1] is well organized, and the length is appropriate.

The title is chosen correctly, and the abstract provides sufficient information to give a clear idea of what to expect from the paper.

The results are well highlighted, and the conclusions are adequate.

The technical depth of the paper meets the requirements for a scientific article published in a quality journal.

## References

[1] Jones RB, Hubble S, Taylor L, Gunn H, Logan A, Rowland T, Bradwell H, Connolly L, Algie k, Anil K, Halliday B, Houston S, Dennett R, Chatfield S, Buckingham S, Freeman J (2021). Technologies to support assessment of movement during video consultations: exploratory study. JMIRx Med.

